# Anthracycline-free or short-term regimen as adjuvant chemotherapy for operable breast cancer: A phase III randomized non-inferiority trial

**DOI:** 10.1016/j.lanwpc.2021.100158

**Published:** 2021-05-13

**Authors:** Ke-Da Yu, Xi-Yu Liu, Li Chen, Miao Mo, Jiong Wu, Guang-Yu Liu, Gen-Hong Di, Claire Verschraegen, Daniel G. Stover, Zhi-Gang Zhuang, François Bertucci, Armando Orlandi, Jie Wang, Giuseppe Lippi, Ke-Jin Wu, Mohammed A. Osman, Lei Fan, Zhi-Ming Shao

**Affiliations:** a Department of Breast Surgery, Fudan University Shanghai Cancer Center, Shanghai, China; b Shanghai Medical College, Fudan University, Shanghai, China; c Shanghai Key Laboratory of Breast Cancer, Shanghai, China; d Department of Cancer Prevention & Clinical Statistics Center, Fudan University Shanghai Cancer Center, Shanghai, China; e Ohio State University Comprehensive Cancer Center, Columbus, OH 43210, United States; f Department of Breast Surgery, Shanghai First Maternity and Infant Hospital, Shanghai, China; g Department of Medical Oncology, Institut Paoli-Calmettes, 13273 Marseille, France; h Comprehensive Cancer Center-Unit of Medical Oncology, Fondazione Policlinico Universitario A. Gemelli IRCCS, Roma, Italy; i Department of Breast Surgery, The International Peace Maternity & Child Health Hospital of China Welfare Institute, Shanghai, China; j Section of Clinical Biochemistry, University Hospital of Verona, Piazzale LA Scuro, Verona 37100, Italy; k Department of Breast Surgery, Obstetrics & Gynecology Hospital of Fudan University, Shanghai, China; l General Organization for Teaching Hospitals, Cairo, Egypt

## Abstract

**Background:**

De-escalating anthracycline is gaining popularity for breast cancer patients. We aim to evaluate the non-inferiority of an anthracycline-free or short-term regimen to the standard anthracycline-based regimen for operable patients with human epidermal growth factor receptor 2 (HER2)-negative breast cancer.

**Methods:**

It is a prospective, open-label, phase 3, randomized non-inferiority trial from June 1, 2010 to June 1, 2017. Follow-up had been kept until July 2019. This trial was conducted at Fudan University Shanghai Cancer Center. Patients with pT1–3N+ or pT2–3N0 but high-risk (grade II/III, lymphovascular invasion, ≤35 years of age or hormone-receptor negative) HER2-negative operable breast cancer were eligible and stratified by age, pathological tumour stage, pathological node status and hormone-receptor status. Patients were randomized to 6 cycles of docetaxel and cyclophosphamide (TC, *n* = 524), 3 cycles of cyclophosphamide/epirubicin/fluorouracil followed by 3 cycles of docetaxel (CEF-T, *n* = 523) or epirubicin and cyclophosphamide for 4 cycles followed by paclitaxel for 12 weeks (EC-P, *n* = 524) as the intention-to-treat population. Of these patients, 94% completed allocated therapy. Difference in disease-free survival (DFS) compared to EC-P. The prespecified non-inferiority margin was 4.5%, corresponding to the hazard ratio (HR) of 1.44 (one-sided α = 0.05), with an assumed 5-year DFS of 89% for EC-P.

**Findings:**

Included in the intention-to-treat population were 1571 patients (median [IQR] age, 50 [45–57] years; 92% estrogen receptor [ER]-positive; 59% pN+). Through a median follow-up of 5.5 years, HR for TC *versus* EC-P was 1.05 (5-year DFS: 85.0% *vs*. 85.9%; 90% confidence interval [CI]: 0.79–1.39, non-inferior *P* = 0.048) and for CEF-T *versus* EC-P, 0.99 (5-year DFS: 85.1% *vs*. 85.9%; 90% CI: 0.75–1.30, non-inferior *P* = 0.045). Grade 3 or 4 adverse events for TC included rash (3.9%) and peripheral neuropathy (2.8%) and for CEF-T and EC-P diarrhea and nausea/vomiting were predominant. Results of per-protocol analyses were similar.

**Interpretation:**

Both TC and CEF-T are non-inferior adjuvant regimen to EC-P mainly in patients with ER+HER2- breast cancer. TC is a safe regimen that avoids anthracycline-related side effects.

**Funding:**

This work was supported by grants from the National Natural Science Foundation of China (Grants 81672600, 81722032, 82072916, and 91959207), the 2018 Shanghai Youth Excellent Academic Leader, the Fudan ZHUOSHI Project, the Municipal Project for Developing Emerging and Frontier Technology in Shanghai Hospitals (grant SHDC12010116), the Cooperation Project of Conquering Major Diseases in the Shanghai Municipality Health System (grant 2013ZYJB0302), the Innovation Team of the Ministry of Education (grant IRT1223), and the Shanghai Key Laboratory of Breast Cancer (grant 12DZ2260100) and the National Cancer Institute (grant P30 CA16058).

**Research in context**

Evidence before this study

Before this study was designed, adjuvant anthracycline-taxane (AT) regimens worked as the standard of care. An increasing number of long-term survivors or elderly patients with operable breast cancer develop heart failure or treatment-related leukemia. De-escalation of anthracycline-containing regimens is gaining popularity.

The US Oncology 9735 reported that 4 cycles of docetaxel and cyclophosphamide (TCx4) was superior to 4 cycles of doxorubicin and cyclophosphamide (ACx4) in terms of disease-free survival (DFS) and overall survival (OS). No evidence supported the non-inferiority of TCx6 compared with EC followed by weekly paclitaxel (EC-P).

CEF-T was developed independently as one of the most effective regimens among all AT-containing regimens. This short-term anthracycline-based regimen has not been directly compared with in patients with HER2-negative breast cancer.

**Added value of this study**

In this study, hazard ratio for TC *versus* EC-P was 1.05 (5-year DFS: 85.0% *vs*. 85.9%; 90% confidence interval [CI]: 0.79–1.39, non-inferior *p* = 0.048) and for CEF-T *versus* EC-P, 0.99 (5-year DFS: 85.1% *vs*. 85.9%; 90% CI: 0.75–1.30, non-inferior *p* = 0.045)**.** Both TC and CEF-T are non-inferior adjuvant regimen to EC-P mainly in patients with ER+HER2- breast cancer.

**Implications of all the available evidence**

This trial will provide evidence about two non-inferior regiments (TC and CEF-T) *versus* EC-P.

Alt-text: Unlabelled box

## Introduction

Adjuvant chemotherapy improves outcomes of operable breast cancer. Since the landmark study of adjuvant chemotherapy with cyclophosphamide, methotrexate, and fluorouracil (1), there has been an ongoing effort to identify better regimens to improve survival and decrease toxicity. A meta-analysis done by the Early Breast Cancer Trialists’ Collaborative Group (EBCTCG) showed the effectiveness of 6-months of anthracycline-based adjuvant regimens for patients with operable breast cancer (2). A later EBCTCG meta-analysis validated the advantage of incorporating taxane into anthracycline-based regimens (3). For the last 20 years, anthracycline and taxane (AT)-based regimens have been the standard adjuvant treatment for operable breast cancer.

However, adjuvant anthracycline chemotherapy showed long-term side effects. An increasing number of long-term survivors or elderly patients with operable breast cancer develop heart failure or treatment-related leukaemia (3). Hence, the de-escalation of anthracycline-containing regimens is gaining popularity in clinical trials. The US Oncology 9735 reported that 4 cycles of docetaxel and cyclophosphamide (TCx4) was superior to 4 cycles of doxorubicin and cyclophosphamide (ACx4) in terms of disease-free survival (DFS) and overall survival (OS) (4). These results promulgated TC as an effective alternative. Since ACx4 was considered a weak comparative regimen, stronger comparators such as taxane plus AC (TaxAC) were studied with TC in later trials. The DBCG 07-READ trial provided no clear evidence of overall benefit from anthracycline for selected operable breast cancer patients with normal copy numbers of the topoisomerase IIα gene (5). The West German Study Group PlanB trial recently demonstrated the non-inferiority of 6 cycles of TC to a standard TaxAC regimen in clinically high-risk or genomically intermediate- to high-risk human epidermal growth factor receptor 2 (HER2)-negative patients (6). All of these promising results encourage researchers to consider switching from anthracycline-based regimens to anthracycline-free regimens in the adjuvant setting of operable breast cancer. Dose-dense anthracycline has been recommended by National Comprehensive Cancer Network (NCCN) guidelines as one of the preferred regimens since 2005 based on the CALGB 9741 trial (7) and the Oxford Overview confirmed a moderate reduction in 10-year recurrence risk and death from breast cancer without increasing the risk of death from other causes by increasing the dose intensity of adjuvant chemotherapy. However, guidelines in China had not recommended the dose-dense AC-P regimen until 2017 due to unbalanced medical resources across China. The dose-dense AC-P regimen was recommended for partially tolerable patients with triple-negative breast cancer. Thus, EC-P was the standard of care in China at the time when this trial was designed.

Besides, three cycles of cyclophosphamide, epirubicin, and fluorouracil followed by three cycles of docetaxel (CEF-T) (8) was developed independently as one of the most effective regimens among all AT-containing regimens. This short-term anthracycline-based regimen has not been directly compared with EC followed by weekly paclitaxel (EC-P) in patients with HER2-negative breast cancer. However, it's worth noting that CEF-T, a preferred regimen when the trial was first designed, is no longer preferred since Del-Mastro et al. (9) and the EBCTCG review (10) reported no benefit from 5-FU since 2015.

This trial, **M**inus **A**nthracycline or **S**hort-**Ter**m *versus* Epirubicin and Cyclophosphamide followed by Paclitaxel Regimen for Adjuvant Breast Cancer Therapy (MASTER), was designed to prospectively test the hypothesis that six cycles of TC are non-inferior to standard AT-containing chemotherapy (EC-P, four cycles of epirubicin and cyclophosphamide followed by weekly paclitaxel). A decision was made to assess, as a secondary outcome, the non-inferiority of short-term anthracycline-based regimen CEF-T to EC-P, after demonstrating the non-inferiority of TC.

## Methods

### Study design

It is a prospective, open-label, phase III, non-inferiority randomized trial of patients with HER2-negative operable breast cancer, approved by the institutional ethics committee of Fudan University Shanghai Cancer Center (FUSCC). The trial was done according to the International Conference on harmonization Good Clinical Practice guidelines and ethical principles in the Declaration of Helsinki. All patients were required to sign an informed consent form before enrollment and randomization.

The trial is registered on ClinicalTrials.gov, identifier: NCT01314833 (https://clinicaltrials.gov/ct2/show/NCT01314833).

It was designed as a three-arm prospective trial to test the non-inferiority of anthracycline-free short-term regimen docetaxel 75 mg/m^2^ and cyclophosphamide 600 mg/m^2^ once every 3 weeks for six cycles (TC) or of short-term anthracycline regimen cyclophosphamide 500 mg/m^2^, epirubicin 100 mg/m^2^, and fluorouracil 500 mg/m^2^ every 3 weeks for 3 cycles followed by docetaxel 100 mg/m^2^ every 3 weeks for 3 cycles (CEF-T), compared with standard long-term AT-containing regimen epirubicin 90 mg/m^2^ and cyclophosphamide 600 mg/m^2^ every 3 weeks for 4 cycles followed by paclitaxel 80 mg/m^2^ weekly for 12 weeks (EC-P) in HER2-negative operable breast cancer.

Assignment to the treatment groups was stratified by age (less than 50 years *vs.* 50 years and older), pathological tumor stage (pT1 *vs.* pT2–3), pathological node status at diagnosis (negative *vs*. positive), and hormone-receptor status (negative *vs*. positive).

### Study population

Eligible patients were women with histologically confirmed, unilateral operable primary invasive breast cancer with known hormone-receptor status, HER2-negative status, and no evidence of metastatic disease by standard laboratory and radiologic testing. Key inclusion criteria were pT1–3 and node-positive (pN+) tumours or pT2–3N0 tumours with at least one of the following risk factors: (1) grade II/III; (2) lymphovascular invasion; (3) ≤35 years of age; (4) estrogen receptor (ER) and progesterone receptor (PR) negative. Patients who had received neoadjuvant therapy (including chemotherapy, radiotherapy, or endocrine therapy) were excluded. Patients with serious active infections, severe organ dysfunction, left ventricular ejection fraction <50%, pregnancy, lactation, or Eastern Cooperative Oncology Group performance status ≥2 were excluded. Inclusion and exclusion criteria are in Supplement 1. Upon completion of treatment, patients underwent follow up surveillance and were scheduled to be seen every 3 months for the first two years and every 6 months after that for 10 years.

Prophylactic use of granulocyte colony-stimulating factor (G-CSF) was scheduled during the first cycle of docetaxel-containing therapy according to the latest versions of NCCN guidelines (11,12). Chemotherapy was administered before radiation therapy if radiation was indicated. Radiotherapy was completed by patients who received breast conservation or with ≥4 involved axillary lymph nodes or those with 1–3 involved axillary lymph nodes along with other high-risk factors. On completion of chemotherapy and/or radiotherapy, endocrine therapy (tamoxifen for premenopausal and aromatase inhibitor for postmenopausal women) was administered to patients with hormone-receptor-positive tumours for 5 years. Bisphosphonates and other drugs affecting bone metabolism were added to patients who are at risk of or have osteoporosis. The trial protocol is included in Supplement 1.

### Endpoints

The primary endpoint was DFS, defined as the time from randomization to occurrence of a new event including local recurrence, regional relapse, distant metastasis, contralateral primary breast cancer, second non-breast invasive cancer (excluding non-melanoma skin cancers), or death from any cause. Patients alive without any predefined event were censored at the time of the last follow-up. Secondary endpoints included (1) distant disease-free survival (DDFS), defined as the time from randomization to the earliest distant metastasis or death from any cause, whichever first; (2) OS, defined as the time from randomization to death from any cause (13); and (3) safety, which was assessed throughout the study treatment according to the Common Terminology Criteria for Adverse Events (CTCAE), version 4.0.

### Statistical analysis

These three cohorts that were included in the efficacy analysis were prespecified in a procedure with a fixed hierarchical sequence to adjust for the type I error rate (14). This trial was designed to assess the non-inferiority of TC *versus* EC-P first, then to assess the non-inferiority of CEF-T *versus* EC-P. Both tests were designed with 80% power at the one-sided alpha of 0.05. The trial assumed a 5-year DFS of 89% for EC-P (15,16). Non-inferiority was defined as the 5-year DFS of TC or CEF-T being not worse than an absolute value of 4.5% below EC-P, corresponding to a limiting hazard ratio (HR) of 1.44. This 4.5% non-inferiority margin was set before starting the trial, following consensus from the trial design group. Under these assumptions, the trial target accrual was approximate 1500 patients, with approximate 200 DFS events expected at the final analysis. HRs were obtained using the Cox proportional hazards model. The upper limit of 90% confidence interval (CI) less than 1.44 was evidence to conclude non-inferiority. Non-inferior P values were calculated according to Gisela Tunes da Silva et al. (17). No interim analysis and only one final analysis were planned for DFS. Because of this, a single terminal hypothesis test with an alpha of 0.05 is applied to the present trial.

All efficacy analyses were performed in the ITT and PP population. Five-year DFS, DDFS, and OS were calculated using the Kaplan–Meier method and were analyzed by the stratified log-rank test. HRs and associated 90% CIs were obtained with the use of a stratified Cox proportional-hazards model, with the study group and stratification factors as covariates. Tests for interaction based on the Cox regression model (treatment × factor) were used to assess the heterogeneity of treatment effects across different subgroups. Forest plots were used to summarize these results. For exploratory subgroup analysis, no multiple testing correction was performed.

Toxicity was assessed in patients who received at least one dose of chemotherapy. The proportion of patients presenting with grade 3 to 4 adverse events in each treatment arm was compared with Fisher’s exact test or X^2^ test, when appropriate.

### Meta-analyses

We used a fixed-effects model based on the logarithm of the HR weighted from individual trials. Cochrane’s Q statistic was utilized to explore statistical heterogeneity between studies. The I^2^ statistic was used to quantify the consistency. In the event of significant heterogeneity (*P* value < 0.05), a random-effect model was used. HRs with the corresponding 95% CIs are presented graphically.

Statistical analysis was performed using Stata version 16.0 software (StataCorp, Texas, USA).

### Role of the funding source

Funders in this trial had no role in study design, data collection, data analysis, interpretation and writing of the report.

## Results

### Patient characteristics

**Fig. 1:**
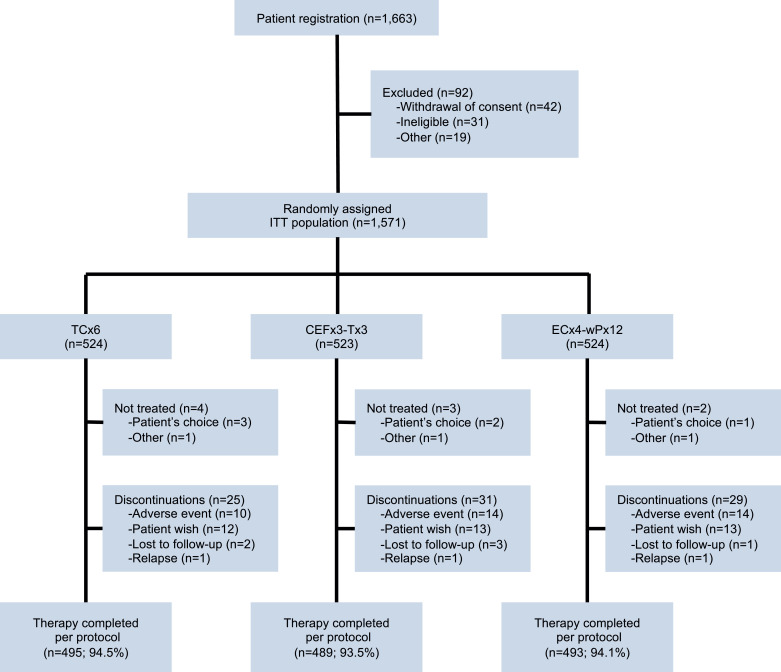
**CONSORT diagram.** Patients registration, exclusions, treatment-arm assignments, and therapy-completion. TCx6: docetaxel 75 mg/m^2^ and cyclophosphamide 600 mg/m^2^ were given once every 3 weeks for six cycles (TC). CEFx3-Tx3: cyclophosphamide 500 mg/m^2^_,_ epirubicin 100 mg/m^2^ and fluorouracil 500 mg/m^2^ were given once every 3 weeks for three cycles followed by docetaxel 100 mg/m^2^ once every 3 weeks for three cycles (CEF-T). ECx4-wPx12: epirubicin 90 mg/m^2^ and cyclophosphamide 600 mg/m^2^ were given once every 3 weeks for four cycles followed by paclitaxel 80 mg/m^2^ once every week for twelve times (EC-P). ITT, intention-to-treat.

A total of 1663 patients were registered between June 2010 and June 2017, and 1571 eligible patients were randomly assigned (1:1:1) to one of three arms following surgery: TC (*n* = 524), CEF-T (*n* = 523) or EC-P (*n* = 524) as the intention-to-treat (ITT) population. Of the ITT population, 94% who completed all cycles of allocated chemotherapy were defined as per-protocol (PP) population: TC (*n* = 495), CEF-T (*n* = 489), and EC-P (*n* = 493), respectively (Fig. 1). Detailed reasons for the exclusion of 92 patients are listed in Table 1 in Supplement 2.

**Table 1:** Baseline characteristics of ITT population.

		TC	CEF-T	EC-P

		*n* = 524 (%)	*n* = 523 (%)	*n* = 524 (%)
Age	<50 years	117 (22.3)	131(25.0)	136 (26.0)
	≥50 years	407 (77.7)	392 (75.0)	388 (74.0)
Menopausal status	Premenopausal	301 (57.4)	289 (55.2)	293 (55.9)
	Postmenopausal	223 (42.6)	234 (44.8)	231 (44.1)
Subtype	Luminal A-like	120 (22.9)	100 (19.1)	117 (22.3)
	Luminal B-like	364 (69.5)	384 (73.4)	366 (69.9)
	TNBC	40 (7.6)	39 (7.5)	41 (7.8)
Histological grade	I-II	350 (66.8)	346 (66.2)	337 (64.3)
	III	174 (33.2)	177 (33.8)	187 (35.7)
Ki-67	≤14%	125 (23.9)	105 (20.1)	120 (22.9)
	>14%	399 (76.1)	418 (79.9)	404 (77.1)
pT	pT1	231 (44.1)	227 (43.4)	237 (45.2)
	pT2–3	293 (55.9)	296 (56.6)	287 (54.8)
pN	pN0	220 (42.0)	212 (40.5)	213 (40.6)
	pN+	304 (58.0)	311 (59.5)	311 (59.4)
Breast surgery	BCS	77 (14.7)	79 (15.1)	72 (13.7)
	Mastectomy	447 (85.3)	444 (84.9)	452 (86.3)
Axillary surgery	SLNB	155 (29.6)	150 (28.7)	152 (29.0)
	ALND	369 (70.4)	373 (71.3)	372 (71.0)
Endocrine therapy	TAM only	159 (30.3)	151 (28.9)	148 (28.2)
	AIs ± OFS only	271 (52.9)	284 (54.3)	275 (52.5)
	TAM to AIs	41 (7.8)	40 (7.6)	45 (8.6)
	None	53 (10.1)	48 (9.2)	56 (10.7)
Radiation therapy	No	302 (57.6)	299 (57.2)	297 (56.7)
	Yes	222 (42.4)	224 (42.8)	227 (43.3)

Abbreviations: AIs, ALND, axillary lymph node dissection; BCS, breast-conserving surgery; CEF-T, cyclophosphamide/epirubicin/fluorouracil followed by docetaxel; EC-P, epirubicin/cyclophosphamide followed by paclitaxel; ITT, intention-to-treat; SLNB, sentinel lymph node biopsy; TC, docetaxel/cyclophosphamide; TNBC, triple-negative breast cancer.

Patient characteristics and baseline clinicopathologic variables were well balanced for the ITT population between both experimental arms (TC or CEF-T) and the control arm (EC-P) (Table 1). The median age was 50 years (interquartile range: 45–57 years); 59% of patients had lymph node-positive disease; 34% had poorly differentiated tumours (grade III). The majority (92.2%) of patients had an estrogen-receptor-positive disease and only 7.6% had triple-negative breast cancer. This is because another concurrent clinical trial carried out competitive recruitment on triple-negative breast cancer in the same period (ClinicalTrials.gov identifier: NCT01216111). Baseline characteristics were well balanced for the PP population as well (Table 2 in Supplement 2).

**Table 2:** Efficacy in the ITT population.

	Arms	Events	Cases	5-yr rate (%)	HR^#^ (90% CI)	Log-rank P*
DFS	TC	72	524	85.0	1.05 (0.79–1.39)	0.771
	CEF-T	73	523	85.1	0.99 (0.75–1.30)	0.946
	EC-P	70	524	85.9	–	–
DDFS	TC	38	524	91.6	0.88 (0.61–1.28)	0.572
	CEF-T	39	523	92.4	0.83 (0.57–1.19)	0.391
	EC-P	43	524	91.4	–	–
OS	TC	21	524	96.5	0.96 (0.58–1.59)	0.893
	CEF-T	24	523	94.9	0.84 (0.51–1.37)	0.549
	EC-P	23	524	95.4	–	–

Abbreviations: CEF-T, cyclophosphamide/epirubicin/fluorouracil followed by docetaxel; CI, confidence interval; DDFS, distant disease-free survival; DFS, disease-free survival; EC-P, epirubicin/cyclophosphamide followed by paclitaxel; HR, hazard ratio; ITT, intention-to-treat; OS, overall survival; TC, docetaxel/cyclophosphamide; yr.: year.

HRs with 90% CIs were calculated using stratified Cox by age (<50 vs. ≥50 years), pT (pT1 *vs*. pT2–3), pN (negative *vs*. positive), and hormone-receptor status (negative *vs*. positive).

⁎ P values were calculated by the stratified log-rank test for comparison with the EC-P arm.

### Efficacy analysis

The median follow-up time was 5.5 years (interquartile range: 3.5–6.7 years). There were 215 DFS events during the follow-up period, of which 72 (13.7%) were in the TC arm, 73 (14.0%) in the CEF-T arm, and 70 (13.4%) in the EC-P arm, respectively. DFS events are summarized in Table 3 in Supplement 2.

**Table 3:** Grade 3 to 4 adverse events.

			TC	CEF-T	EC-P	TC *vs.* EC-P	CEF-T *vs.* EC-P			
			*N* = 524	%	*N* = 523	%	*N* = 524	%	*p*-value	*P*-value
Dose reduction	41	7.9	53	10.2	59	11.2	0.058	0.556		
Dose delay	2	0.5	2	0.5	10	1.9	0.038	0.038		
Blood and lymphatic system disorders										
	Neutropenia	290	55.3	279	53.4	284	54.1	0.710	0.782	
	Anemia	4	0.8	5	0.9	7	1.4	0.547	0.773	
	Febrile neutropenia	11	2.0	9	1.7	10	1.9	0.826	0.820	
	Thrombocytopenia	1	0.2	1	0.2	2	0.3	1.000	1.000	
GI disorders										
	Diarrhea	18	3.5	38	7.2	37	7.0	0.008	0.898	
	Mucositis/stomatitis	2	0.3	7	1.4	9	1.7	0.064	0.617	
	Nausea/vomiting	19	3.6	50	9.5	55	10.5	<0.001	0.614	
Cardiac disorders										
	Ejection fraction decreased	1	0.2	5	0.9	6	1.1	0.124	1.000	
	Ventricular arrhythmia	2	0.3	5	0.9	7	1.2	0.178	0.773	
	Heart failure	0	0.0	0	0.0	1	0.2	1.000	1.000	
Hepatobiliary disorders										
	AST increase	10	1.9	11	2.0	10	1.9	1.000	0.822	
	ALT increase	7	1.3	6	1.1	6	1.1	0.780	1.000	
	Hepatic failure	0	0.0	0	0.0	1	0.2	1.000	1.000	
General										
	Allergy	7	1.3	6	1.1	4	0.8	0.547	0.547	
	Edema	3	0.6	2	0.5	2	0.3	1.000	1.000	
	Fatigue	24	4.6	24	4.5	26	5.0	0.772	0.777	
	Pain	7	1.3	7	1.4	13	2.5	0.176	0.177	
	Fever	2	0.5	2	0.3	3	0.6	1.000	0.655	
	Rash	21	3.9	18	3.4	8	1.6	0.014	0.046	
Infection										
	Wound infection	2	0.5	3	0.6	3	0.6	1.000	1.000	
	Pulmonary infection	2	0.3	2	0.5	3	0.6	1.000	1.000	
	Urinary infection	1	0.2	0	0.0	1	0.2	1.000	1.000	
Hand & Foot										
	Thrombosis	1	0.2	1	0.2	0	0.0	1.000	0.500	
	Peripheral neuropathy	15	2.8	4	0.8	3	0.6	0.007	0.726	
Cardiac-related death*	1	0.2	0	0.0	1	0.2	1.000	1.000		
Acute myeloid leukemia*	0	0.0	0	0.0	1	0.2	1.000	1.000		

⁎ During follow-up; ALT, alanine transaminase; AST, aspartate transaminase; CEF-T, cyclophosphamide/epirubicin/fluorouracil followed by docetaxel; EC-P, epirubicin/cyclophosphamide followed by paclitaxel; GI: gastrointestinal; TC, docetaxel/cyclophosphamide.

Five-year DFS for ITT population treated with TC or EC-P were 85.0% *vs.* 85.9%, respectively (HR=1.05; 90% CI, 0.79–1.39; non-inferior *P* = 0.048) (Table 2). Since the prespecified margin for non-inferiority was 1.44, the 90% CI upper limit of 1.39 was conclusive that TC is non-inferior to EC-P. In ER+ population, five-year DFS for TC or EC-P were 85.7% *vs.* 85.9% (HR=0.99; 90% CI, 0.74–1.33) (Table 4, Supplement 2). Since the prespecified margin for non-inferiority was 1.44, the 90% CI upper limit of 1.39 was conclusive that TC is non-inferior to EC-P.

**Fig. 2:**
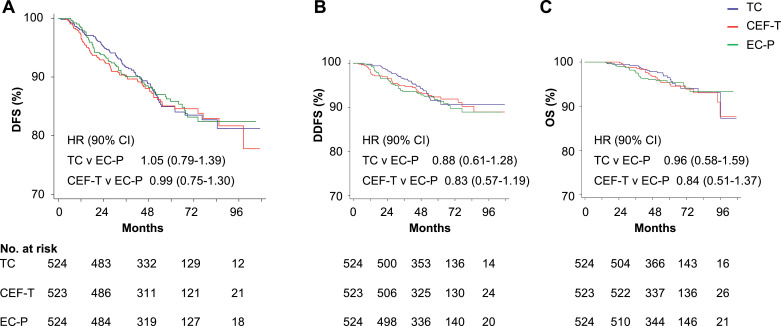
DFS, DDFS and OS in ITT population In the intention-to-treat (ITT) population, Kaplan–Meier curves for (A) disease-free survival (DFS), (B) distant recurrence-free survival (DDFS), and (C) overall survival (OS) of each arm were illustrated. Hazard ratios (HR) with 90% confidence intervals (CIs) were calculated based on the stratified Cox model. Numbers at risk were as listed below figures. CEF-T, cyclophosphamide/epirubicin/fluorouracil followed by docetaxel; EC-P, epirubicin, and cyclophosphamide followed by paclitaxel; TC, docetaxel, and cyclophosphamide.

Then we evaluated the non-inferiority for CEF-T *versus* EC-P, the 5-year DFS were 85.1% *versus* 85.9%, respectively (HR=0.99; 90% CI, 0.75–1.30, non-inferior *p* = 0.045). The criterion for the non-inferiority of CEF-T to EC-P was achieved as well. Moreover, there were no significant differences in DFS, OS, or DDFS between the two experimental arms (TC and CEF-T) and the control arm (EC-P) (Fig. 2).

The PP analysis yielded equivalent results. The HR for TC *versus* EC-P was 1.05 (90% CI, 0.79–1.39), and for CEF-T *versus* EC-P, 0.99 (90% CI, 0.75–1.30) (Table 5 in Supplement 2). Survival curves can be found in Fig. 1 in Supplement 2.

**Fig. 3:**
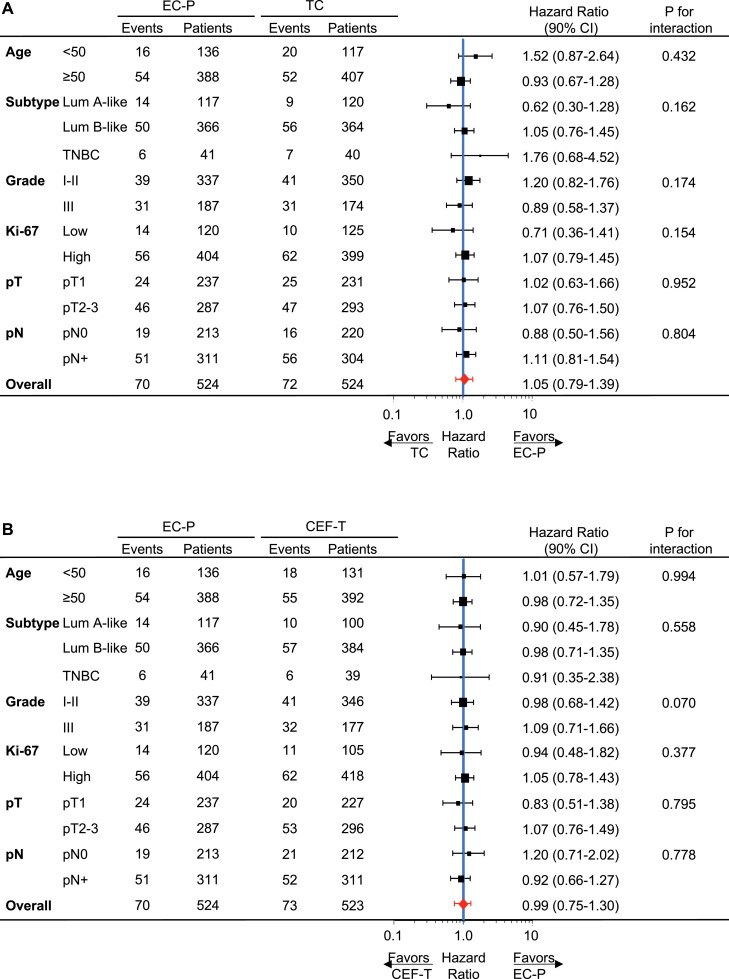
Forest plot for DFS hazard ratios in subgroups by ITT analysis In the intention-to-treat (ITT) population, hazard ratios (HRs) were calculated within each subgroup. (A) TC *vs.* EC-P, HR>1 favours EC-P; (B) CEF-T *vs.* EC-P, HR>1 favours EC-P. CEF-T, cyclophosphamide/epirubicin/fluorouracil followed by docetaxel; EC-P, epirubicin, and cyclophosphamide followed by paclitaxel; Lum A-like: Luminal-A like; Lum B-like: Luminal-B like; pN: pathological node status; pT, pathological tumour stage.

Fig. 3 shows a forest plot of the HR with 90% CI for DFS in major subgroups by ITT analysis (HR >1 would favour EC-P). Subgroup analysis showed similar treatment effects by histological grade, Ki-67, tumour stage and nodal status, but suggested differential effects by age and breast cancer subtype between EC-P and TC. EC-P appears to be more effective than TC in patients with triple-negative breast cancer. Besides, in a subgroup with age < 50 years, EC-P showed a better effect. A similar forest plot by PP analysis is illustrated in Fig. 2 in Supplement 2. Kaplan–Meier curves for each tumour subtype in Fig. 3, Supplement 2.

### Toxicity

Adverse events were recorded on patients who received at least one dose of allocated chemotherapy (Table 3). No treatment-related death occurred during treatment. Compared with the TC arm, the EC-P arm was characterized by significantly more frequent dose delays [TC *versus* EC-P: 2 (0.5%) *versus* 10 (1.9%); *p* = 0.038]. The use of G-CSF prophylaxis during the first cycle of docetaxel-containing therapy was documented in 83% and 86% of patients in the TC and CEF-T arms, respectively. Incidences of grade 3–4 neutropenia, febrile neutropenia and hepatic dysfunctions were similar between arms. Noteworthy grade 3 or 4 adverse events specific to TC were rash (*n* = 21, 3.9%) and peripheral neuropathy (*n* = 15, 2.8%). The rate of peripheral neuropathy is similar to the rates previously reported in the PATTERN trial (4) and CBCSG-010 trial (5) in our centre. Besides, WSG Plan B trial reported 0.8–2.2% of grade 3–4 peripheral neuropathy as well. EC-P arm exhibited more gastrointestinal (GI) disorders, like nausea/vomiting (*n* = 55, 10.5%), diarrhea (*n* = 37, 7.0%), and mucositis/stomatitis (*n* = 9, 1.7%), which may made patients feel more uncomfortable subjectively. In the CEF-T arm, fewer dose delays, but a significantly higher frequency of grade 3 to 4 rash were observed, compared to the EC-P arm. Cardiac toxicity was infrequent in all arms. Grade 3 or 4 cardiac events for TC, CEF-T, and EC-P included ejection fraction decrease (*n* = 1, 0.2%; *n* = 5, 0.9%; *n* = 6, 1.1%), ventricular arrhythmia (*n* = 2, 0.3%; *n* = 5, 0.9%; *n* = 7, 1.2%), and heart failure (0%; 0%; *n* = 1, 0.2%), respectively. During additional follow-up, two deaths (one in TC, one in EC-P arm) were observed as a result of heart failure. One case of acute myeloid leukaemia was observed in the EC-P arm.

### Meta-analysis

We performed a meta-analysis by a fixed-effects model, which included 8 RCTs that compared TC with anthracycline-containing therapy, namely HORG (18), ABC (19), DBCG 07-READ (5), WSG Plan *B* + SUCCESS C combined analysis and this MASTER trial (eFigure 4 in Supplement 2). The total number of patients is 14,312 patients. All the patients were HER2-negative operable breast cancer. By the fixed effects model, the total effect for TC *versus* A+T regimens was estimated. Overall, a non-statistically significant increase in the hazard of 7% was observed between TC and A+T in DFS, with a 95% confidence interval ranging from a 3% reduction to a 19% increase (HR 1.07, 95% CI 0.97–1.19).

## Discussion

In this trial, both adjuvant TC and CEF-T met the non-inferior standard to EC-P mainly in patients with ER+/HER2- operable breast cancer. The 90% upper confidence limit of the HR *versus* EC-P for TC was 1.39 and for CEF-T was 1.30 by ITT analysis, which were both below the non-inferiority boundary of 1.44 with both non-inferior *p* values less than 0.05. There were fewer cardiac events and fewer side effects with TC, yielding a favourable therapeutic index for the anthracycline-free regimen.

To our knowledge, this MASTER trial is the second large randomized non-inferiority study supporting TC as a potential anthracycline-free regimen. The PlanB trial demonstrated the non-inferiority of six cycles of TC to EC-T (4 cycles of epirubicin and cyclophosphamide followed by 4 cycles of docetaxel) in clinically high-risk or genomically intermediate- to high-risk patients (6). Baseline characteristics in our cohort showed a higher node-positive rate and larger tumour size compared with that of the PlanB trial. The 5-year DFS for TC and EC-T in PlanB reached 90%, while in the MASTER trial, the 5-year DFS for TC and EC-P are slightly lower. In contrast, two other randomized trials did not show that TC is non-inferior to the TaxAC standards. The HORG trial, designed as a non-inferiority trial, aimed to compared TCx6 to dose-dense CEF-T (18). This trial included only 650 patients and used a non-inferiority margin of 7% for a 3-year DFS, which might have inadequate power to show non-inferiority. The ABC pooled analysis also failed to report non-inferiority of TCx6 compared to TaxAC (19). That trial enrolled more pN+ and triple-negative diseases than our trial. This trial set an original non-inferiority margin of HR at 1.18. However, the median follow-up was limited to 3.3 years. 4-year DFS rates were 88.2% for TCx6 and 90.7% for TaxAC, yielding an HR of 1.23 (95% CI, 1.01–1.50). Explanations for differences between these four studies include baseline characteristics, sample size, and a conservative non-inferiority margin with an unplanned shortened observation period in the ABC trial. A meta-analysis has summarized these randomized controlled trials comparing TC *versus* AT-containing regimens in HER2-negative operable breast cancer and no differences were observed for DFS and OS (20). We also performed a meta-analysis with the MASTER trial included. Overall, for TC *versus* anthracycline-containing therapy, a non-statistically significant increase in the hazard was observed. The upper boundary of 95% CI is 1.19, indicating TC as an acceptable regimen for HER2-negative operable breast cancer. Six cycles of TC shorten the treatment term but prolong the taxane duration, which might explain the non-inferiority of TCx6 to TaxAC.

This trial is also the first study supporting the non-inferiority of CEF-T to EC-P. CEF-T regimen represents a compromise option, with 20% dose reduction and 20% duration saving, which offers an alternative anthracycline-based regimen. Patients received CEF-T showed fewer dose delay than EC-P, indicating higher compliance during the procedure for a shortened AT-based regimen.

No patients died from treatment-related death. With routine prophylactic G-CSF treatment, the incidence of grade 3 or 4 neutropenia and febrile neutropenia is similar for all groups. As to the GI toxicity, six cycles of TC regimen exhibited less dose delay, and fewer grade 3–4 vomiting and nausea events. TC was easier to be accepted and finished by patients. Anthracycline-related acute cardiac toxicity includes a higher incidence of decreased ejection fraction in the EC-P arm. Only one case of congestive heart failure was reported in the EC-P arm and none in the TC arm, consistent with previous trials (4,6,19). In summary, the acute toxicity profile favours TC. Moreover, though we have not systematically reported long-term cardiac disease, TC showed fewer heart-related disorders than EC-P during treatment. Besides, most patients in our centre came from various places across China and received outpatient chemotherapy. Fewer cycles of chemotherapy mean less travelling and accommodation costs. Though dose-dense EC followed by dose-dense paclitaxel shortens the chemo to a great deal, it is still not routinely operated in China because of lacking enough supportive care.

The following limitations should be considered when interpreting our results. First, this open-label study was performed in a single centre with all patients who were Chinese, although the results of our trial are expected to apply to patients in Western countries. Thus, cautions are needed for ethnic extrapolation. Considering biases might be introduced by open-label studies, several measures were taken, *e.g.* setting objective endpoints, following up patients by an independent team, and cleaning basic data by independent statisticians. Second, long-term cardiac disease and treatment-related leukaemia are being collected in the long-term follow-up. Limited cases were observed at this time point. Continuous follow-up has been kept until the preset 10 years to follow OS events as well as to better summarize long-term side effects. Third, our trial was designed 10 years ago. The 4.5% non-inferiority margin was originally set according to clinical status and contemporary other trials at that time. For example, earlier in 2009, the WSG Plan B trial designed their trial with a non-inferiority margin of 4.4% for 5-year DFS. The 4.5% non-inferiority margin seems not as suitable as expected for the current status. However, it was complicated to adjust the prespecified non-inferiority boundary after patient inclusion completion. Besides, there is a gap between the actual observed survival rate (85.9%) and the preset survival rate (89%) for the EC-P arm. Fourth, clinical trials about adjuvant treatment require a large sample size and years of observation to draw meaningful conclusions, which often lag innovative diagnostic features and novel therapies. The RxPONDER study (21) recently demonstrated no need for adjuvant chemotherapy in the genomically low risk (recurrence score ≤25) postmenopausal population with 1–3 positive lymph nodes. Future clinical trials in our centre will combine molecular subtyping and modern predictive biomarkers for generating more precise therapeutic guidance and identify those who may gain substantial benefit from anthracycline. Finally, we must admit that though there is great interest in patients with triple-negative disease, limited cases were identified due to competitive enrollment populations. Though subgroup analysis by tumour subtype showed various results, the subgroup analysis is underpowered due to the small number of cases. One thing to note is that a large proportion of patients in our cohort are with ER+ breast cancer, thus the conclusion that TC and CEF-T are non-inferior to EC-P is mainly applicable in those with ER+/HER2- breast cancer.

In conclusion, our clinical outcome findings suggest that TC for 6 cycles as well as CEF-T are effective adjuvant chemotherapy mainly for patients with ER+/HER2- breast cancer.

## Contributors

Conception and design: Ke-Da Yu, Lei Fan, Zhi-Ming Shao

Financial support: Ke-Da Yu, Zhi-Ming Shao

Provision of study material or patients: All authors

Collection and assembly of data: All authors

Data analysis and interpretation: All authors

Manuscript writing: All authors

Final approval of manuscript: All authors

Accountable for all aspects of the work: All authors

## Declaration of Competing Interest

The authors have declared no conflicts of interest.

## Appendix Supplementary materials

Image, application 1

Image, application 2

Image, application 3
