# Supplementary material for: Anthracycline-free or short-term regimen as adjuvant chemotherapy for operable breast cancer: A phase III randomized non-inferiority trial
Source: Lancet Reg Health West Pac. 2021 May 13;11:100158. doi: 10.1016/j.lanwpc.2021.100158 (PMC8315472; doi:10.1016/j.lanwpc.2021.100158)
Supplement: Supplementary file 1 [file mmc1.docx]

**Trial Protocol**

**List of Contents:**

1. Inclusion Criteria
2. Exclusion Criteria
3. Dose and schedule
4. Dose modifications
5. Endpoints
6. Sample Calculation
7. Pathologic evaluation method
8. Supplementary References

**1. Inclusion Criteria:**

- Written informed consent
- Female patients, age at diagnosis 18-75 years
- Histological confirmed unilateral primary invasive carcinoma of the breast
- Adequate surgical treatment with complete resection of the tumour (R0) and resection of >or =10 axillary nodes or sentinel lymph node (SLN) in clinically node negative (N0) patients
- Human epidermal growth factor receptor 2 (HER2) negative judged by two pathologists according to updated guidelines of the American Society of Clinical Oncology/College of American Pathologists (ASCO/CAP)^1,2^
- Node positive disease or node negative disease with at least one other risk factor (tumour size >or =2 cm, grade ≥II)
- No evidence for distant metastasis (M0) after conventional staging
- Eastern Cooperative Oncology Group (ECOG) performance status≤1
- The patient must be accessible for treatment and follow-up
- Negative pregnancy test (urine or serum) within 7 days prior to randomization in premenopausal patients
- Adequate organ and bone marrow function as evidenced by

1. Leucocytes ≥4 x 10^9^/L
2. Platelets ≥100 x 10^9^/L
3. Hemoglobin ≥9 g/dL
4. Total bilirubin ≤1.5 UNL
5. Aspartate transaminase (AST) and alanine transaminase (ALT) ≤2.5 UNL
6. Creatinine <175 mmol/L (2 mg/dL)

- Left ventricular ejection fraction (LVEF) >50%

**2. Exclusion Criteria**

- Has received neoadjuvant therapy (include chemotherapy, targeted therapy, radiotherapy or endocrine therapy)
- Has bilateral breast cancer
- Has previous history of additional malignancy, with the exception of adequately treated basal cell carcinoma and cervical carcinoma in situ
- Has metastatic (Stage 4) breast cancer
- Has any >T4 lesion (UICC1987) (with skin involvement, mass adhesion and fixation, and inflammatory breast cancer)
- Pregnant, or breast feeding, or women of childbearing age who cannot practice effective contraceptives
- Patients participating in other clinical trials at the same time
- Has severe organ dysfunction (cardiopulmonary liver and kidney) insufficiency, LVEF <50% (cardiac ultrasound); severe cardio cerebral vascular disease within the 6 months previous of randomization (such as unstable angina, chronic heart failure, uncontrolled hypertension with blood pressure >150/90mmHg, myocardial infarction, or cerebral blood vessel); diabetic patients with poor blood glucose control; patients with severe hypertension
- Has known allergy to taxanes and excipients
- Has severe or uncontrolled infection
- Inability to comply with study and follow‐up procedure
- Any other finding giving reasonable suspicion of a disease or condition that contraindicates the

**3. Dose and schedule:**

**Arm 1**: 6 cycles of TC (Docetaxel 75mg/m^2^ ivgtt d1 + Cyclophosphamide 600 mg/m^2^ iv d1, 21 days per cycle).

**Arm 2**: 3 cycles of CEF (epirubicin 100 mg/m^2^ ivgtt d1 + Cyclophosphamide 500 mg/m^2^ iv d1 + 5-fluorouracil 500 mg/m^2^ iv d1, 21 days per cycle) followed by 3 cycles of Docetaxel (Docetaxel 100mg/m^2^, ivgtt d1, 21 days per cycle)

**Arm 3**: 4 cycles of EC (epirubicin 90 mg/m^2^ ivgtt d1 + Cyclophosphamide 600 mg/m^2^ iv d1, 21 days per cycle) followed by 4 cycles of Paclitaxel (Paclitaxel 80mg/m^2^, ivgtt d1, 8, 15, 21days per cycle).

Radiotherapy is indicated to patients who received breast conservation or with ≥4 involved axillary lymph nodes or those with 1-3 involved axillary lymph nodes along with other high-risk factors. On completion of chemotherapy ± radiotherapy, endocrine therapy is administered to patients with hormone-receptor-positive tumours for 5 years. G-CSF can be used for treatment purpose or as a prophylactic routine according to updated versions of National Comprehensive Cancer Network (NCCN) guidelines on myeloid growth factors^3,4^. Dexamethasone, cimetidine, or promethazine can be used prior to paclitaxel or docetaxel as preventive medication. The administration of other cytotoxic drugs is not permitted for all participants during allocated treatment. The administration of novel drugs for chronic diseases should inform the investigator.

**4. Dose modifications:**

**4.1 Treatment interrupted or aborted**

In the following scenario, patients should discontinue their allocated treatment:

- Medical conditions that are harmful to patients' health judged by investigators
- Unacceptable toxicity, for example: more than twice dose delay and/or dose reduction due to hematologic toxicity reduction twice; grade 3 or 4 non-hematologic toxicity occurs for the third time
- Patients still cannot receive the designated treatment for 42 days after the last administration of previous cycle
- Patient requirements
- Relapse of the disease
- Poor compliance

If a patient withdraws from the study treatment, every effort should be made to keep up-to-date on the annual survival status, to get the latest survival status for at least 5 years after the enrollment, and to make effective efforts to determine the reason why the patient cannot follow up or withdraw from the trial. If the patient stops the treatment or follow-up, detailed cause must be recorded and the patient who withdrew from the trial cannot be replaced.

**4.2 Dose reduction**

Dose reduction is allowed according to the severest level of overall toxicity. Drug reduction due to hematological or non-hematologic toxic reactions is permanent.

- Hematological toxicity

Dose reductions caused by hematological toxicity should be based on the lowest levels of neutrophils and platelet counts tested after the previous cycle, following the table below:

Table 1. Dose adjustment during the cycle based on the minimum count of neutrophils caused by the previous cycle

| **Neutrophils**  **(×10^9^/L)** | **Dose of docetaxel** | **Dose of paclitaxel** | **Dose of epirubicin** | **Dose of cyclophosphamide** |
| --- | --- | --- | --- | --- |
| > 1.5 | 100% | 100% | 100% | 100% |
| 1.0-1.49 | 75% | 75% | 75% | 75% |
| < 1.0 | Dose delay | Dose delay | Dose delay | Dose delay |

- Non-hematological toxicity

Dose reduction due to non-hematologic toxicity will be implemented following the tables below:

Table 2. Dose adjustment during the cycle based on the non-hematological toxicity

| **Toxicity** | **Grade** | **Dose of docetaxel** | **Dose of paclitaxel** | **Dose of epirubicin /cyclophosphamide** |
| --- | --- | --- | --- | --- |
| **Liver** | 0-2 | 100% | 100% | 100% |
|  | 3 | 50% | 50% | 50% |
|  | 4 | Dose delay | Dose delay | Dose delay |
| **Myalgia or peripheral neuropathy** | 0-1 | 100% | 100% | 100% |
|  | 2 | 50% | 50% | 50% |
|  | 3/4 | Dose delay | Dose delay | Dose delay |
| **Mucositis** | 0-2 | 100% | 100% | 100% |
|  | 3 | 50% | 50% | 50% |
|  | 4 | Dose delay | Dose delay | Dose delay |
| **Bradycardia with symptoms** | Any | Dose delay | Dose delay | Dose delay |
| **Other toxicity (except nausea, vomiting and hair loss)** | 0-2 | 100% | 100% | 100% |
|  | 3 | 50% | 50% | 50% |
|  | 4 | Dose delay | Dose delay | Dose delay |

**5. Endpoints**

The primary endpoint is disease-free survival (DFS), defined as the time from randomization to the occurrence of the earliest event as follows: local recurrence, regional recurrence, distant recurrence, contralateral second primary invasive breast cancer, second non-breast invasive cancer (excluding non-melanoma skin cancers), or death from any cause. Patients alive without any predefined event are censored at the time of the last follow-up. Secondary endpoints included distant disease-free survival (DDFS) (defined as the time from randomization to the earliest recurrence outside of the ipsilateral locoregional region or to death from any cause, whenever a death occurred before distant recurrence) and overall survival (OS) (defined as the time from randomization to death from any cause). Safety is also a secondary endpoint and is assessed throughout the study treatment according to the Common Terminology Criteria for Adverse Events (CTCAE), version 4.0.

**6. Sample Calculation:**

The sample size is calculated on the basis of the primary hypothesis. This trial is designed to assess the non-inferiority of the TC *versus* EC-P first and next the non-inferiority of the CEF-T *versus* EC-P in a procedure with a fixed hierarchical sequence to adjust for the type I error rate^5^. The trial assumes a 5-year DFS of 89% for EC-P. Non-inferiority is defined as the 5-year DFS of TC or CEF-T being not worse than an absolute value of 4.5% below EC-P, following consensus from the trial design group. To achieve the non-inferiority hypothesis at one-sided significance level of 5% and power of 80%, allocation to three arms will require approximately 1,500 patients and 200 events in total. Hazard ratios will be obtained using the stratified COX proportional hazards model. An upper limit of 90% CI less than 1.44 is evidence to conclude non-inferiority. The Independent Data Monitoring Committee (IDMC) the MASTER steering committee takes the responsibility to monitor, analyze and report the final results.

**7. Pathologic evaluation method:**

Primary surgically removed tumour tissues were sent to the Department of Pathology in Fudan University Shanghai Cancer Center (FUSCC) for slide review, immunohistochemistry (IHC) staining, and fluorescence *in situ* hybridization (FISH) analysis. Two experienced breast pathologists assessed histology and central grade and were both blinded to the clinical data and to Ki67 expression. Slides were stained for estrogen receptor (ER) (rabbit [SP1]; Neomarkers, Fremont, CA), progesterone receptor (PR) (mouse monoclonal PgR636; DAKO, Glostrup, Denmark), and Ki67 (clone 30-9 rabbit monoclonal; Ventana, Tucson, AZ) using standard protocols. Tumours were classified as ER or PR positive if IHC was present in ≥1% of tumour nuclei. Ki67 was evaluated in at least 100 tumour cells within the high-density area semi-quantitatively (in 5% increments) and quantitatively (in 1% increments). Additionally, the patients with HER2 expression status (IHC, score =2) were subjected to fluorescence in situ hybridization (FISH) screening for HER2 gene amplification. The HER2 negative subgroup was defined as FISH negative with IHC score<3 or IHC score<2.

**8. Supplementary References:**

1. Wolff AC, Hammond ME, Schwartz JN, et al. American Society of Clinical Oncology/College of American Pathologists guideline recommendations for human epidermal growth factor receptor 2 testing in breast cancer. *J Clin Oncol.* 2007;25(1):118-145.

2. Wolff AC, Hammond ME, Hicks DG, et al. Recommendations for human epidermal growth factor receptor 2 testing in breast cancer: American Society of Clinical Oncology/College of American Pathologists clinical practice guideline update. *J Clin Oncol.* 2013;31(31):3997-4013.

3. NCCN Clinical Practice Guidelines in Oncology, Myeloid Growth Factors, Version 2.2014. [*http://wwwnccnorg*](http://wwwnccnorg)*.* 2014.

4. Crawford J, Becker PS, Armitage JO, et al. Myeloid Growth Factors, Version 2.2017, NCCN Clinical Practice Guidelines in Oncology. *J Natl Compr Canc Netw.* 2017;15(12):1520-1541.

5. Westfall PH, Krishen A, Young SS. Using prior information to allocate significance levels for multiple endpoints. *Stat Med.* 1998;17(18):2107-2119.
