# Supplementary material for: Anthracycline-free or short-term regimen as adjuvant chemotherapy for operable breast cancer: A phase III randomized non-inferiority trial
Source: Lancet Reg Health West Pac. 2021 May 13;11:100158. doi: 10.1016/j.lanwpc.2021.100158 (PMC8315472; doi:10.1016/j.lanwpc.2021.100158)
Supplement: Supplementary file 2 [file mmc2.docx]

**Supplement 2**

**List of Contents:**

1. eTables
2. eFigures

**1. Supplementary Tables:**

**eTable 1. Detailed reasons for excluded patients**

|  | **Reasons** | **No. of patients (%)** |
| --- | --- | --- |
| **Withdrawal of consent** |  | **42 (45.7)** |
|  | Quit adjuvant treatment | 4 (4.3) |
|  | Return to a local hospital for treatment | 32 (34.8) |
|  | Private affairs | 6 (6.5) |
| **Ineligible** |  | **31 (33.7)** |
|  | Ductal carcinoma in situ | 9 (9.8) |
|  | Previous invasive disease | 4 (4.3) |
|  | Bilateral breast cancer | 3 (3.3) |
|  | Severe organ dysfunction (atrial fibrillation/ renal insufficiency/ liver insufficiency/…) | 5 (5.4) |
|  | Received neoadjuvant therapy | 2 (2.2) |
|  | Eastern Cooperative Oncology Group performance status ≥2 | 8 (8.7) |
| **Other** |  | **19 (20.7)** |
|  | Joined other trials | 11 (12.0) |
|  | Loss of contact | 8 (8.7) |
| **Total** |  | **92 (100.0)** |

**eTable 2. Baseline characteristics of the PP population.**

|  |  | **TC** | | **CEF-T** | | **EC-P** | |
| --- | --- | --- | --- | --- | --- | --- | --- |
|  |  | ***N*=495** | **%** | ***N*=489** | **%** | ***N*=493** | **%** |
| **Age** | **<50 years** | 113 | 22.8 | 126 | 25.8 | 130 | 26.4 |
|  | ≥**50 years** | 382 | 77.2 | 363 | 74.2 | 363 | 73.6 |
| **Menopausal status** | **Premenopausal** | 291 | 58.8 | 276 | 56.4 | 281 | 57.0 |
|  | **Postmenopausal** | 204 | 41.2 | 213 | 43.6 | 212 | 43.0 |
| **Subtype** | **Luminal A** | 113 | 22.8 | 93 | 19.0 | 110 | 22.3 |
|  | **Luminal B** | 347 | 70.1 | 359 | 73.4 | 343 | 69.6 |
|  | **TNBC** | 35 | 7.1 | 37 | 7.6 | 40 | 8.1 |
| **Histological grade** | **I-II** | 332 | 67.1 | 321 | 65.6 | 313 | 63.5 |
|  | **III** | 163 | 32.9 | 168 | 34.4 | 180 | 36.5 |
| **Ki-67** | **≤14%** | 117 | 23.6 | 98 | 20.0 | 113 | 22.9 |
|  | **>14%** | 378 | 76.4 | 391 | 80.0 | 380 | 77.1 |
| **pT** | **pT1** | 220 | 44.4 | 209 | 42.7 | 220 | 44.6 |
|  | **pT2-3** | 275 | 55.6 | 280 | 57.3 | 273 | 55.4 |
| **pN** | **pN0** | 207 | 41.8 | 196 | 40.1 | 193 | 39.1 |
|  | **pN+** | 288 | 58.2 | 293 | 59.9 | 300 | 60.9 |
| **Breast surgery** | **BCS** | 70 | 14.1 | 74 | 15.1 | 70 | 14.2 |
|  | **Mastectomy** | 425 | 85.9 | 415 | 84.9 | 423 | 85.8 |
| **Axillary surgery** | **SLNB** | 149 | 30.1 | 140 | 28.6 | 142 | 28.8 |
|  | **ALND** | 346 | 69.9 | 349 | 71.4 | 351 | 71.2 |

**Abbreviations**: ALND, axillary lymph node dissection; BCS, breast-conserving surgery; CEF-T, cyclophosphamide/epirubicin/fluorouracil followed by docetaxel; EC-P, epirubicin/cyclophosphamide followed by paclitaxel; ER, estrogen receptor; PP, per-protocol; SLNB, sentinel lymph node biopsy; TC, docetaxel/cyclophosphamide

**eTable 3. First invasive disease-free survival event**

|  | **TC** | **CEF-T** | **EC-P** | **Total** |
| --- | --- | --- | --- | --- |
| **Type of event** | **n=524** | **n=523** | **n=524** | **n=1,571** |
| **Recurrence** |  |  |  |  |
| **Local** | 9 | 13 | 13 | 35 |
| **Distant** | 27 | 29 | 33 | 89 |
| **Contralateral breast cancer** | 4 | 3 | 4 | 11 |
| **Other secondary primary cancer** | 29 | 26 | 17 | 72 |
| **Death** | 3 | 2 | 3 | 8 |
| **Total** | 72 | 73 | 70 | 215 |

**Abbreviations**: CEF-T, cyclophosphamide/epirubicin/fluorouracil followed by docetaxel; EC-P, epirubicin/cyclophosphamide followed by paclitaxel; TC, docetaxel/cyclophosphamide

**eTable 4. Efficacy in the ER+ ITT population**

|  | **Arms** | **Events** | **Cases** | **5-yr rate (%)** | **HR^#^ (90% CI)** | **Log-rank P*^*^*** |
| --- | --- | --- | --- | --- | --- | --- |
| **DFS** | TC | 64 | 483 | 85.7 | 0.99 (0.74-1.33) | 0.953 |
|  | CEF-T | 67 | 483 | 85.2 | 0.99 (0.75-1.30) | 0.946 |
|  | EC-P | 64 | 483 | 85.9 | - | - |
| **DDFS** | TC | 32 | 483 | 92.3 | 0.83 (0.51-1.33) | 0.434 |
|  | CEF-T | 35 | 483 | 92.6 | 0.86 (0.58-1.27) | 0.525 |
|  | EC-P | 37 | 483 | 91.9 | - | - |
| **OS** | TC | 16 | 483 | 96.8 | 0.78 (0.40-1.51) | 0.454 |
|  | CEF-T | 21 | 483 | 95.1 | 0.87 (0.47-1.62) | 0.664 |
|  | EC-P | 20 | 483 | 95.6 | - | - |

**Abbreviations:** CEF-T, cyclophosphamide/epirubicin/fluorouracil followed by docetaxel; CI, confidence interval; DDFS, distant disease-free survival; DFS, disease-free survival; EC-P, epirubicin/cyclophosphamide followed by paclitaxel; ER, estrogen receptor; HR, hazard ratio; ITT, intention-to-treat; OS, overall survival; TC, docetaxel/cyclophosphamide; yr.: year.

HRs with 90% CIs were calculated using stratified Cox by age (<50 *vs.* ≥50 years), pT (pT1 *vs.* pT2-3), pN (negative *vs*. positive), and hormone-receptor status (negative *vs*. positive).

* P values were calculated by the stratified log-rank test for comparison with the EC-P arm.

**eTable 5. Efficacy test in PP population**

|  | **Arms** | **Events** | **Cases** | **5-yr rate (%)** | **Hazard Ratio^#^** | **90% CI** | **Log-rank P^*^** |
| --- | --- | --- | --- | --- | --- | --- | --- |
| **DFS** | TC | 66 | 495 | 85.3 | 1.05 | 0.79-1.39 | 0.776 |
|  | CEF-T | 67 | 489 | 85.1 | 0.99 | 0.75-1.30 | 0.959 |
|  | EC-P | 64 | 493 | 86.2 | - | - | - |
| **DDFS** | TC | 34 | 495 | 91.9 | 0.87 | 0.55-1.39 | 0.563 |
|  | CEF-T | 34 | 489 | 92.8 | 0.80 | 0.50-1.27 | 0.335 |
|  | EC-P | 39 | 493 | 91.6 | - | - | - |
| **OS** | TC | 18 | 495 | 96.6 | 0.94 | 0.49-1.80 | 0.854 |
|  | CEF-T | 20 | 489 | 95.8 | 0.82 | 0.44-1.55 | 0.539 |
|  | EC-P | 20 | 493 | 96.0 | - | - | - |

**Abbreviations:** CEF-T, cyclophosphamide/epirubicin/fluorouracil followed by docetaxel; CI, confidence interval; DDFS, distant disease-free survival; DFS, disease-free survival; EC-P, epirubicin/cyclophosphamide followed by paclitaxel; PP, per-protocol; OS, overall survival; TC, docetaxel/cyclophosphamide; yr.: year.

^#^ Hazard ratio was calculated using stratified Cox by age (<50 *vs.* ≥50 years), pT (pT1 *vs.* pT2-3), pN (negative *vs*. positive), and hormone-receptor status (negative *vs*. positive).

* P values were calculated by the stratified log-rank test for comparison with the EC-P arm.

**2. Supplementary Figures:**

**eFigure 1:** DFS, DDFS, and OS by PP analysis.

In the per-protocol (PP) population, Kaplan-Meier curves for (A) disease-free survival (DFS), (B) distant recurrence-free survival (DDFS), and (C) overall survival (OS) of each arm were illustrated. Hazard ratios with 90% confidence intervals were calculated based on the stratified Cox model. Numbers at risk were as listed below figures.

CEF-T, cyclophosphamide/epirubicin/fluorouracil followed by docetaxel; EC-P, epirubicin/cyclophosphamide followed by paclitaxel; TC, docetaxel and cyclophosphamide.

**eFigure 2:** Forest plot for DFS hazard ratios in subgroups by PP analysis.

In the per-protocol (PP) population, (A) TC *vs.* EC-P, hazard ratio (HR) >1 favours EC-P; (B) CEF-T *vs.* EC-P, HR >1 favours EC-P.

CEF-T, cyclophosphamide/epirubicin/fluorouracil followed by docetaxel; EC-P, epirubicin/cyclophosphamide followed by paclitaxel; pN, pathological node status; PP, per-protocol; pT, pathological tumour stage; TC, docetaxel and cyclophosphamide.

Unadjusted HR with 90% confidence interval (CI) was calculated by the stratified univariate proportional hazards model.

**eFigure 3:** Kaplan-Meier curves for disease-free survival (DFS) by subtypes in (A) intention-to-treat analysis or (B) per-protocol analysis.

Hazard ratios with 90% confidence intervals (CI) were calculated based on Cox model.

Numbers at risk were as listed below figures.

TC: Docetaxel and cyclophosphamide once every 3 weeks for six cycles.

CEF-T: Cyclophosphamide, epirubicin and fluorouracil once every 3 weeks for three cycles followed by docetaxel once every 3 weeks for three cycles.

EC-P: Epirubicin and cyclophosphamide once every 3 weeks for four cycles followed by paclitaxel once every week for twelve times.

**eFigure 4:** Forest plots and the pooled hazard ratios in the overall population by fixed-effects model

Abbreviations: HR, hazard ratio; CI, confidence intervals; TC, docetaxel and cyclophosphamide; A+T, anthracycline and taxane-based chemotherapy
